# Role of weather and other factors in the dynamics of a low‐density insect population

**DOI:** 10.1002/ece3.9261

**Published:** 2022-09-06

**Authors:** Christer Solbreck, Jonas Knape, Jonas Förare

**Affiliations:** ^1^ Department of Ecology Swedish University of Agricultural Sciences Uppsala Sweden

**Keywords:** climate change, density dependence, Noctuidae, population dynamics, weather effects

## Abstract

Insect population dynamics are the result of an interplay between intrinsic factors such as density dependence, trophic web interactions, and external forces such as weather conditions. We investigate potential mechanisms of population dynamics in a natural, low‐density insect population. Eggs and larvae of the noctuid moth, *Abrostola asclepiadis*, develop on its host plant during summer. The population density, and mortality, was closely monitored throughout this period during 15 years. Densities fluctuated between one and two orders of magnitude. Egg–larval developmental time varied substantially among years, with lower survival in cool summers with slower development. This was presumably due to the prolonged exposure to a large guild of polyphagous arthropod enemies. We also found a density‐dependent component during this period that could be a result of intraspecific competition for food among the last larval instars. Dynamics during the long period from pupation in late summer through winter survival in the ground to adult emergence and oviposition the next year displayed few clear patterns and more unexplained variability, thus giving a more random appearance. The population hence shows more unexplained or unpredictable variation during the long wintering period, but seems more predictable over the summer egg–larval period. Our study illustrates how weather—via a window of exposure to enemies and in combination with density‐dependent processes—can determine the course of population change through the insect life cycle.

## Graphical abstract

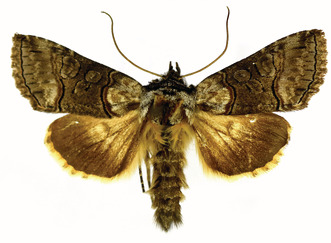

## 1 INTRODUCTION

Weather conditions are important factors in the dynamics of insect populations. Among the various weather factors known to affect insects, temperature conditions are of particular importance in temperate regions. Insects are ectotherms, and cool weather limits population growth. Temperature *directly* affects many aspects of insect life such as growth rates, survival probability, reproductive rates, and flight propensity (Andrewartha & Birch, 1954; Bale et al., 2002). In addition to these direct effects, weather conditions also impact insect populations *indirectly* through the web of trophic interactions (Abarca & Spahn, 2021). For example, plant resources used by phytophagous insects are often affected by weather (DeLucia et al., 2012; Hambäck, 2021; Solbreck & Knape, 2017), thus impacting the food–herbivore link. Interactions via the natural enemy link may also be modified by weather conditions (Barton & Ives, 2014; Barton & Schmitz, 2009; Pepi et al., 2018).

Population dynamics are the result of this interplay between intraspecific processes, trophic web interactions and external forcing from weather conditions. Hence, weather effects should not be considered in isolation but in combination with other factors, particularly density‐dependent trophic interactions (Royama, 1992; Varley et al., 1973). The combined effects of weather and web are often complex resulting in a wide range of dynamical responses depending on the specific circumstances (Ives, 1995; Klapwijk et al., 2012; Lawson et al., 2015; Stenseth et al., 2002; Uszko et al., 2017; Walther, 2010). Due to these complexities, we still have a poor understanding how weather and future climates will affect insect population change, motivating a continued analysis of specific population systems.

Holometabolous insects have complex life cycles, and ecological effects during each life history phase may be unique (Kingsolver et al., 2011). Each phase can be seen as a time window dominated by specific interactions. Window widths wax and wane in response to environmental conditions, with sometimes strong effects on survival. For example, the slow‐growth high‐mortality theory, summarized by Benrey and Denno (1997), postulates that slow growth causes longer exposure to enemies resulting in higher mortality risks. Although this theory was originally proposed for insects with different development times caused by food quality changes, the same effect can be expected from developmental differences due to different weather regimes. It was also shown experimentally that temperature‐induced slow growth resulted in higher enemy‐induced mortality (Benrey & Denno, 1997).

However, showing that a larger time window of exposure to enemies increases mortality is not enough to explain change in natural populations, particularly so in insects with ecologically different life history stages. What is happening during one life history phase may be modified by processes during other stages. A grasp of processes affecting the *entire* life cycle is essential when analyzing how weather conditions affect population change (Ådahl et al., 2006; Radchuk et al., 2013). This also requires a prior strong selection of variables based upon knowledge of biological and ecological conditions (Knape & de Valpine, 2011).

We analyze the dynamics of a low‐density, non‐outbreak population of a noctuid moth. The larva is monophagous, feeding on the leaves of a patchily distributed perennial herb. Earlier studies have indicated that natural enemies take a heavy toll on eggs and larvae and that this mortality is higher during cool summers (Förare, 1995b). In other words, it appeared that during cold summers the time window of exposure to enemies widened causing higher mortality. Here, we develop a Bayesian population model to investigate the time window effects in relation to other processes—such as density‐dependent effects and winter weather conditions—affecting the insect population through its entire life cycle. Previous studies are also extended by using a much larger data set (15 years compared with 6 years in the previous study).

## 2 MATERIALS AND METHODS

### 2.1 Biology

**FIGURE 1:**
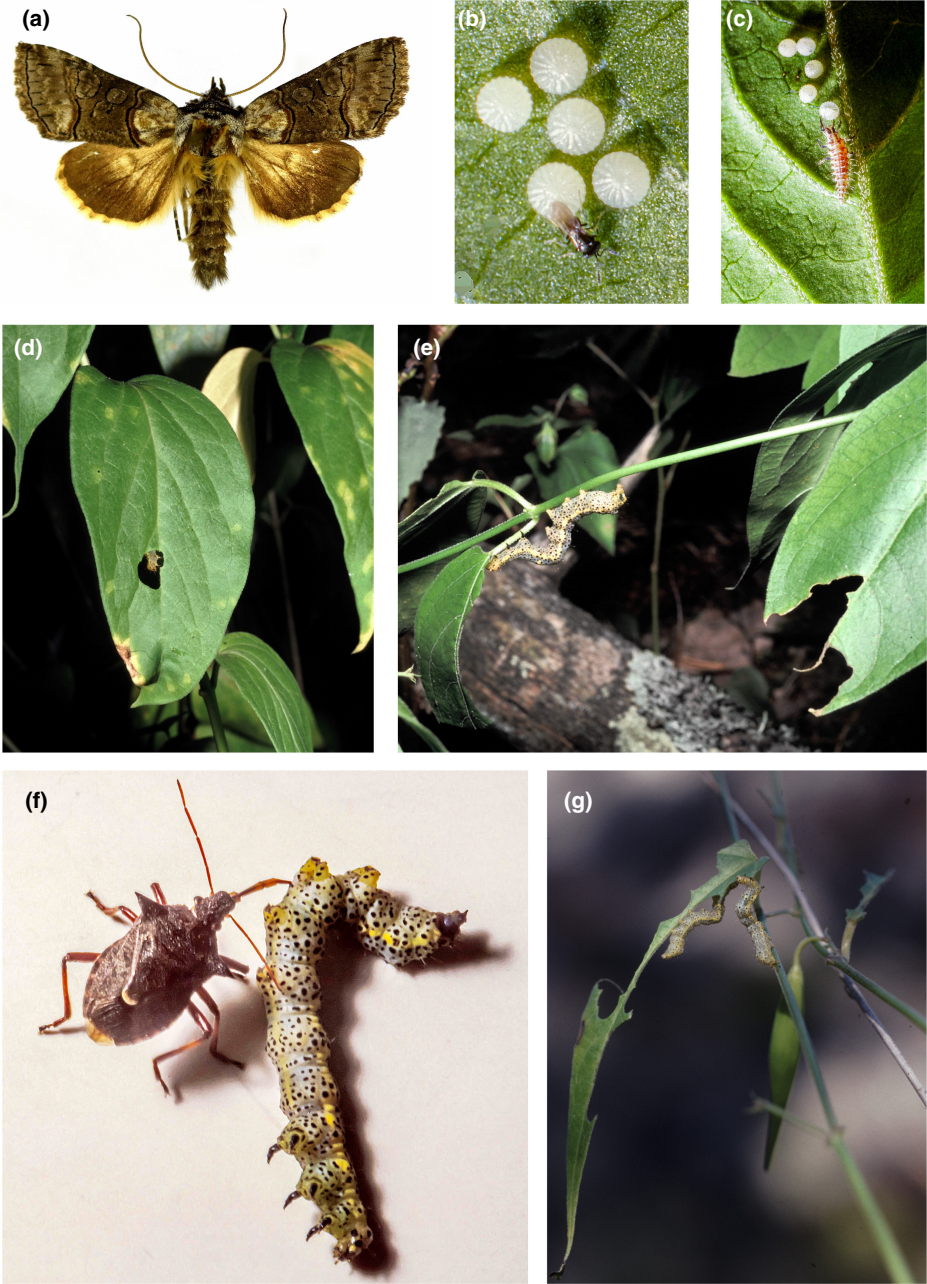
(a) Adult *Abrostola asclepiadis* moth. (b) Egg batches on the underside of *V. hirundinaria* leaves attacked by undetermined Hymenoptera parasitoid, and (c) by predatory lacewing (Neuroptera) larva. (d) Third instar larva chewing a hole in a leaf and (e) a fifth (last) instar larva chewing large chunks off leaves. (f) Predatory bug (*Picromerus bidens*) (Pentatomidae) with a newly killed last instar larva. (g) Two last instar larvae competing for remaining leaf late in summer. Photographs by Bert Gustafsson (a), Jonas Förare (b, c, f), and Christer Solbreck (d, e, g).

**FIGURE 2:**
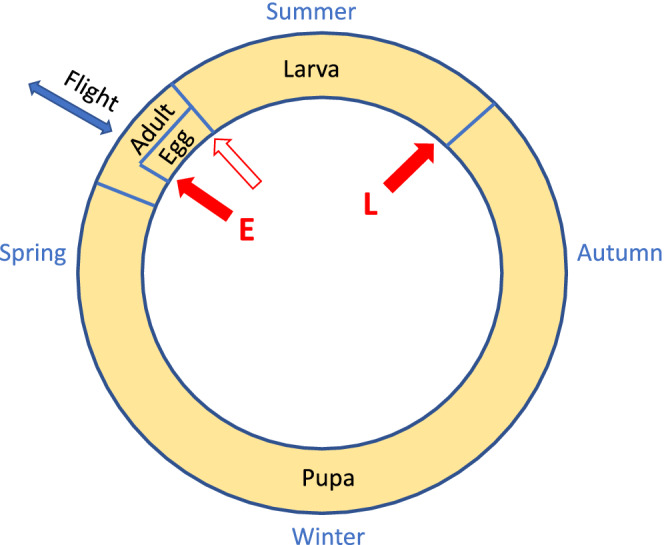
Life cycle of *A. asclepiadis*. Egg–larval development takes place on the host plant during June–August (September). When larvae are mature, they wander some meters and enter the soil where they pupate inside cocoons. They remain in the soil for about nine months. Adult moths emerge in late May–June. They are strong fliers frequently moving between host plant patches. Red arrows indicate life stages monitored: the number of eggs laid and hatched and the number of last instar larvae. The filled arrows indicate the two stages (E and L) used in the main statistical analysis.

The larva of *Abrostola asclepiadis* Schiff. (Lepidoptera, Noctuidae) is monophagous on the long‐lived herb, white swallow‐wort, *Vincetoxicum hirundinaria* Med. (Apocynaceae) (Figure 1a,d,e). The insect has a southeastern distribution in Sweden, which closely follows that of its host plant. It has one generation per year in Sweden (Figure 2). Moths fly in early summer with a peak in June. Females deposit eggs in small batches (usually 1–5 eggs) on the underside of *V. hirundinaria* leaves (Figure 1b,c). Females are good flyers and are capable of laying more than 200 eggs. They will deposit numerous egg batches in several host plant patches. Short host plant individuals growing in shaded positions are preferred for oviposition (Förare, 1995b; Förare & Engqvist, 1996; Förare & Solbreck, 1997).

Eggs hatch after about 10 days, and larvae need another 5 weeks to develop. Average development time is thus 6–7 weeks, but there is considerable variation among years depending on temperature conditions (see below). Larvae of the first two instars are active day and night whereas older larvae (instars 3–5) (Figure 1d,e) are mainly night‐active. Young larvae feed collectively making small holes in the central parts of leaves. Instar 4 and 5 larvae concentrate their feeding on young leaves at the top of the plants. They feed singly and consume large parts of entire leaves (Figure 1e). They are difficult to find during daytime when they are hiding, but are easy to spot at night with the aid of a torch. When full‐grown, larvae leave the host plant to pupate in the soil, where they remain as pupae until adult emergence early the next summer.

Several kinds of invertebrate enemy attack eggs and larvae of *A. asclepiadis*, but they are all generalists. Eggs are attacked by two species of Hymenoptera parasitoids (*Trichogramma* sp and *Telenomus* sp) (Figure 1b) as well as by many species of polyphagous predators including ants, chrysopid larvae (Figure 1c), anthocorid bugs, and mites. The same kinds of predators also attack young larvae. The pentatomid bug *Picromerus bidens* (L.) attacks larger larvae (Figure 1f). Tachinid and braconid parasitoids have been reported from older larvae elsewhere, but they are very rare in the study area. Hundreds of larvae brought to the laboratory for other experiments never yielded any parasitoids. No vertebrate predators have ever been observed, and pathogens are very rarely observed (Förare, 1995b). Because the later larval instars are night‐active and hide during the day, bird predation—which may be hard to directly observe—is unlikely to be of significance.

### 2.2 Study area and host plant

The study area at Tullgarn (58°57′N, 17°36′E) is situated on the coast about 50 km SSW of Stockholm. Populations of the host plant have a distinctly patchy distribution in this landscape (Solbreck, 2012). Plant individuals typically form dense tussocks of from a few up to more than 100 40–80 cm tall flowering shoots. The main flowering period is June–July. The host plant is poisonous (Kalske et al., 2014; Tullberg et al., 2000), and its community of phytophagous insects is very small. *A. asclepiadis* is the only leaf‐feeding insect on the plant in the study area (except for rare stray specimens of polyphagous Lepidoptera species). Three species feed on flowers or seeds. There is one flower gall midge *Contarinia vincetoxici* Kieffer (Diptera, Cecidomyiidae) and two seed predator species, *Euphranta connexa* (Fabr.) (Diptera, Tephritidae) and *Lygaeus equestris* (L.) (Heteroptera, Lygaeidae) (Solbreck & Knape, 2017; Widenfalk et al., 2002) attacking the plant.

### 2.3 Sampling

Four plots were monitored 1990–2004. They had 326 ± 67, 712 ± 187, 743 ± 159, and 924 ± 114 (mean ± SD for entire study period) shoots of *V. hirundinaria*. All plots were within a 4 km distance. The plots were inspected once every week during egg and larval periods (usually early June to mid‐August). The eggs were counted by inspecting the underside of every leaf for the presence of *A. asclepiadis* eggs. The position of every egg batch was marked. During later visits, the fates of all eggs were noted. Eggs were classified into four groups: (1) hatched, (2) parasitized (blackened), (3) predated (disappeared or sucked out or with chewing holes different from the openings of hatched eggs), and (4) unhatched and dead.

Last instar larvae (stage V) were also censused weekly. They were counted at night when larvae are active and easy to spot in light of a torch. The search was guided by observations of leaf damage and larval droppings, as well as by earlier observations of the positions of IV instar larvae. When a last instar larva was encountered, it was marked with a felt pen so as not to be double‐counted on later visits. Egg and larval totals were calculated for each plot and year (Appendix S1). For a detailed discussion of measurement accuracies, see Appendix S3. We do not have direct data on predation of larvae.

The short census intervals allow us to determine times of egg or larval appearances and to calculate the length of the egg–larval development period for each year. Egg–larval development time was calculated as the difference between the date of the first observed egg and the first observed last instar larva (in any of the plots). The reason for using first observations of eggs/larvae rather than mean or median times is that they are easily observed. There are generally no single eggs or larvae appearing well before the others. Furthermore, because eggs are deposited and large larvae are formed over a long period of time, mean/median dates of deposition become more variable and less accurate.

### 2.4 Weather factors

Our choice of weather factors to be considered in the analysis is based upon previously published studies (Förare, 1995a, 1995b; Förare & Engqvist, 1996; Förare & Solbreck, 1997) as well as on 10 additional years of field observations (C. Solbreck, unpublished).

#### 2.4.1 The egg–larval development period (summer period)

Temperature is a prime weather factor affecting eggs and larvae. Laboratory rearings had shown a very tight relationship between temperature and larval development rate for the range of temperatures encountered in the field (Förare & Engqvist, 1996). We, therefore, proceed in two steps when analyzing temperature effects. First, we investigate the effect of mean daily temperature during the development period on development time (using linear regression), and second, we include the effect of development time in a population model for survival of larvae (see population model below). We alternatively explored a model with only a direct temperature effect (no effect of development time) and a model with both a direct effect of temperature and an indirect effect via development time.

Development takes place during different periods in different years (first observed egg ranged from June 7 to July 8, and first fifth instar larva observed by varied by almost two months July 14 to September 10) (Figure 4), and we calculated mean temperature for the specific days of the observed development period each year.

#### 2.4.2 The pupal to adult period (winter period)

We envisaged two possible weather factors that might affect pupal survival: (1) Winter minimum temperature. Very low temperatures could potentially cause freezing of pupae. (2) The duration of the period with snow‐covered ground. This might, for example, facilitate predation on pupae by winter active arthropod predators or small mammals. On the contrary, snow cover acts as an insulation against cold and it could potentially shield pupae from winter lows.

The final brief period, encompassing adult emergence and flight, may also be affected by temperature. For example, high temperatures are likely to provide more nights with good flight conditions, resulting in more eggs laid. In our analysis, we used average May–June air temperature.

In summary, the following environmental factors were analyzed. For the egg–larva period (“summer period”): mean temperature during egg–larval development (“summer temperature”), egg–larval development time, and a combination of the two. For the (“winter period”): Winter minimum temperature, number of days with snow cover, and mean air temperature for May–June. All weather data were obtained from the standard meteorological station in Stockholm, about 50 km to the north of the study area.

### 2.5 Data treatment and population model

We pooled data from the four patches because most of the individuals come from a single patch, and the remaining patches had too few individuals to reliably fit our population model below. For reference, estimates from separate analyses of data from single patches are provided in Table 1. Insect abundance was measured at three points in the life cycle of *A. asclepiadis*, namely eggs laid, eggs hatched (=first instar larvae produced), and fifth instar larvae produced (Figure 2).

**TABLE 1:** Parameter estimates of the models.

Parameter	Parameter meaning	Estimate (95% HPD interval)	Pooled data, with winter covariates	Pooled data with spring covariate	Pooled data, with summer temperature, without development time	Pooled data, with summer temperature and development time	Data from patch T40A only	Data from patch T23 only	Data from patch T14 only	Data from patch T9 only
*a*	Intercept for hazard rate	3.8 (1.9, 5.8)	3.7 (1.7, 5.7)	3.7 (1.8, 5.5)	−2.8 (−5.14, −0.55)	4.2 (−1.5, 8.9)	3.4 (1.0, 5.8)	0.0 (−3.1, 4.1)	2.9 (−1.7, 7.6)	6.4 (3.1, 9.5)
*b*	Slope for “density dependence”	−0.20 (−0.32, −0.07)	−0.20 (−0.32, −0.07)	−0.20 (−0.31, −0.07)	−0.06 (−0.24, 0.11)	−0.21 (−0.37, −0.03)	−0.18 (−0.33, −0.02)	−0.12 (−0.31, 0.07)	−0.61 (−1.00, −0.25)	−0.31 (−0.47, −0.13)
*c*	Slope for development time	−1.0 (−1.5, −0.6)	−1.0 (−1.5, −0.5)	−1.0 (−1.4, −0.6)		−1.0 (−1.7, −0.2)	−1.0 (−1.5, −0.4)	−0.3 (−1.4, 0.6)	−0.6 (−1.9, 0.6)	−1.7 (−2.4, −0.9)
*σ*_ε_	SD for random year effect	0.16 (0.09, 0.27)	0.16 (0.09, 0.27)	0.16 (0.09, 0.26)	0.29 (0.18, 0.45)	0.17 (0.09, 0.30)	0.18 (0.10, 0.30)	0.11 (0.00, 0.59)	0.10 (0.00, 0.47)	0.18 (0.02, 0.41)
	Slope for summer temp on larval survival				0.10 (0.01, 0.20)	−0.01 (−0.11, 0.11)				
*μ*	Intercept	4.6 (4.0, 5.3)	4.4 (2.7, 6.0)	3.1 (0.6, 5.7)	4.6 (4.0, 5.3)	4.6 (4.0, 5.2)	4.6 (3.8, 5.4)	3.2 (2.0, 4.4)	1.9 (0.9, 2.8)	4.3 (3.2, 5.4)
*f*	Slope for “density dependence”	−0.41 (−0.66, −0.17)	−0.43 (−0.74, −0.12)	−0.50 (−0.76, −0.23)	−0.41 (−0.65, −0.15)	−0.41 (−0.65, −0.16)	−0.43 (0.80, −0.09)	0.28 (−2.56, 3.05)	−0.62 (−2.57, 1.37)	−0.62 (−1.28, 0.07)
*σ*_η_	SD for random year effect	0.49 (0.33, 0.76)	0.52 (0.34, 0.83)	0.47 (0.32, 0.73)	0.49 (0.33, 0.77)	0.49 (0.33, 0.74)	0.60 (0.39, 0.96)	0.93 (0.32, 2.18)	1.06 (0.53,1.99)	0.86 (0.52, 1.52)
	Slope for winter min temp		−0.03 (−0.13, 0.06)							
	Slope for days of snow cover		−0.003 (−0.016, 0.010)							
	Slope for spring temperature			0.13 (−0.08, 0.34)						

*Note*: The first column shows estimates for the base model that does not include environmental covariates, and where data are pooled across the four patches. The next two columns show estimates for pooled data with additional covariates for the winter period from larvae to eggs. In the first of these, the covariates are winter minimum temperature and the number of days with snow cover. In the second, there is a covariate for spring temperature, which might affect the number of flying adults laying eggs. The next two columns show results from models with direct effects of average temperature during development on larval survival, when development itself is not included and when it is included. The last four columns show estimates for the base model fitted separately to data from each patch.

For the population model, we just used two of the annual measures, namely the egg stage (E) soon after oviposition, and the final larval stage (L) approximately 1–2 months later (Figure 2). We thus model the population process in two steps per year. The first encompasses most of egg–larval stages, and the second (mainly) the pupa–adult stages until oviposition.

Patterns in egg predation and parasitization were investigated visually (Appendix S2).

#### 2.5.1 Survival during egg–larval period

Given that there were *E*_*t*_ eggs in the beginning of the season in year *t*, we model survival to the final larval stage using a binomial model

$$L_{t}∼\text{Binomial}\;\left(E_{t},s_{t}\right)$$

where *s*_*t*_ is the probability of survival over the entire period. The binomial distribution accounts for demographic stochasticity in survival. We model the survival probability as a function of the observed annual development time, the number of eggs laid to account for possible density dependence, and a random year effect to account for additional environmental stochasticity. We introduce these variables using a log–log link for the probability of eggs to survive until the final larval stage:

(1) $$s_{t}=\exp\left(-\exp\left(-\left(a+b\;\log\;E_{t}+c\;\log\;d_{t}+\;\varepsilon_{t}\right)\right)\right)$$

where *a* is an intercept, *b* a slope for density dependence, *d*_*t*_ is the estimated development time in year *t* and *c* its slope coefficient, and *ε*_*t*_ is a normally distributed random year effect. In additional models, an extra term for direct temperature effects was included, and development time left out or kept, as previously described. The choice of a log–log link and the inclusion of the logarithm of development time for survival implies that the survival probability *s*_*t*_ corresponds to the survival probability up to time *d*_*t*_ under a Weibull hazard rate (Pinder et al., 1978). This hazard is a power function of time with shape determined by the parameter *c* (*c* = 1 corresponds to a constant hazard) and scale determined by the other covariates and the random effect.

#### 2.5.2 Pupal–adult survival to oviposition

The second part of the model involves the process from (the latter part of) last instar larvae in the autumn of year t to the number of eggs laid the following year. Thus, it may be seen as a simple model of the combined effect of several subprocesses in the development from the final larval stage, through the overwintering pupal and emerging adult stages. The per capita productivity is modeled linearly on the log scale with an intercept term, a slope for the log number of larvae describing density dependence, and a random year effect. To account for demographic stochasticity in productivity, we use Poisson distributions. The second submodel, therefore, is

(2) $$E_{t}∼\text{Poisson}\left(L_{t-1}\;\exp\left(\mu +f\;\log\;L_{t-1}+\eta_{t}\right)\right)$$

where *μ* is an intercept, *f* a slope for density effects, and *η*_*t*_ is a random year effect. In this base model, no weather effects are included, but we additionally fit two models, one with winter weather (minimum temperature and number of snow days) and one with spring temperature (May and July) as covariates in the exponent of Equation (2), Table 1.

We fitted models in a Bayesian framework using MCMC sampling via the JAGS software (Plummer, 2017) using 4 chains and 2 million iterations. All parameters were given vague prior distributions (uniform(−10, 10) for fixed effect coefficients and scaled Cauchy distributions for standard deviations of random effects). Convergence of MCMC chains was assessed through visual inspection of parameter traceplots and via r‐hat metrics, which were below 1.01 for all model parameters. The code for the analysis is provided in Data S1 and S2.

## 3 RESULTS

**FIGURE 3:**
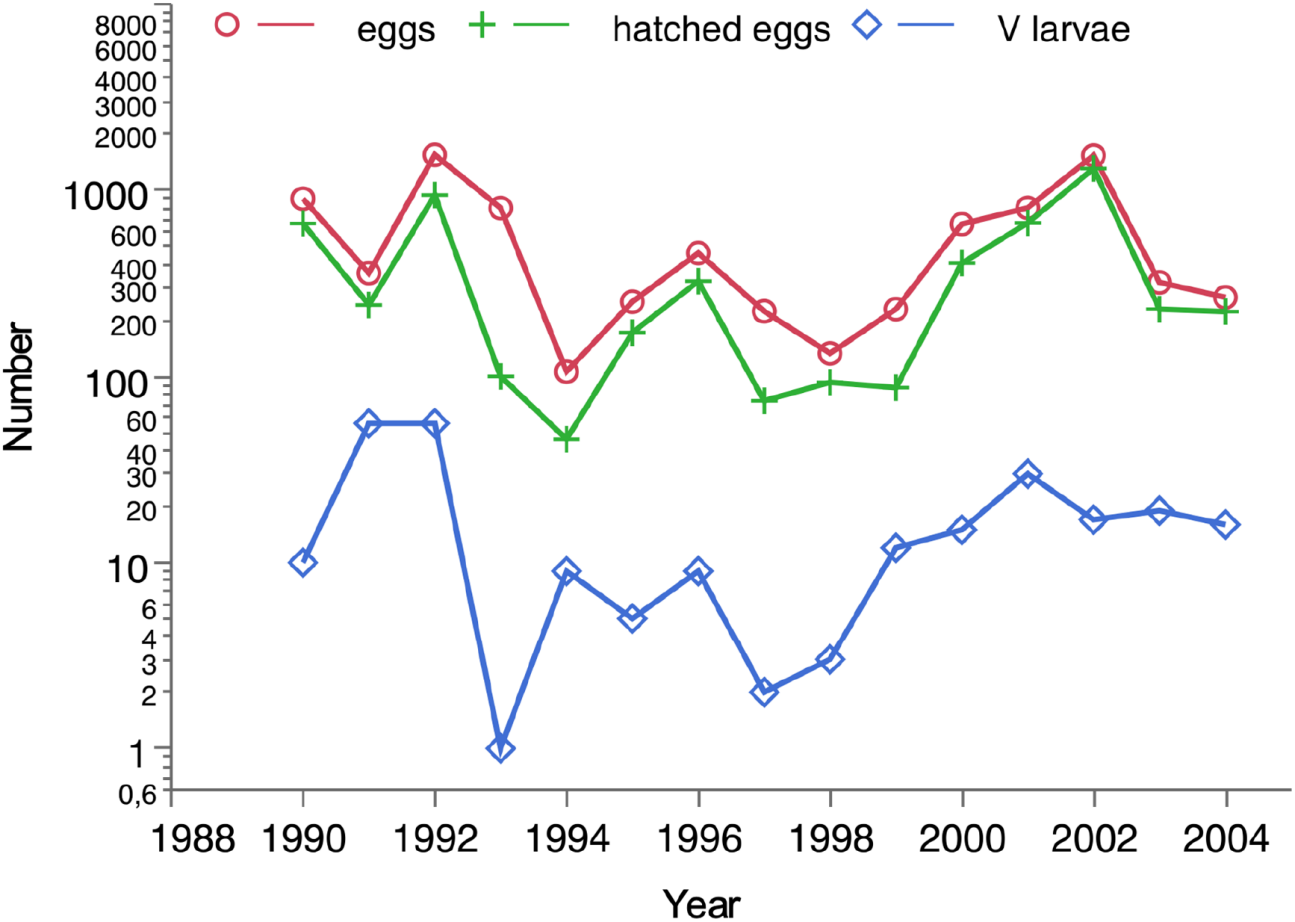
Numbers of eggs laid, eggs hatched, and last instar larvae of *A. asclepiadis* 1990–2004.

The range of population fluctuations in egg and larval abundances was between one and two orders of magnitude (Figure 3). The mortality was higher during the larval stages than during the egg stage. Almost all mortality during the egg stage was due to arthropod predators and Hymenoptera parasitoids (Appendix S1). Egg predation and parasitization showed no clear temporal trends, but there was a potential negative association between egg predation and temperature (Appendix S2). Larval parasitoids and pathogens are very rare in the study area and predators seem to be the dominating enemies of larvae (see Section 4).

**FIGURE 4:**
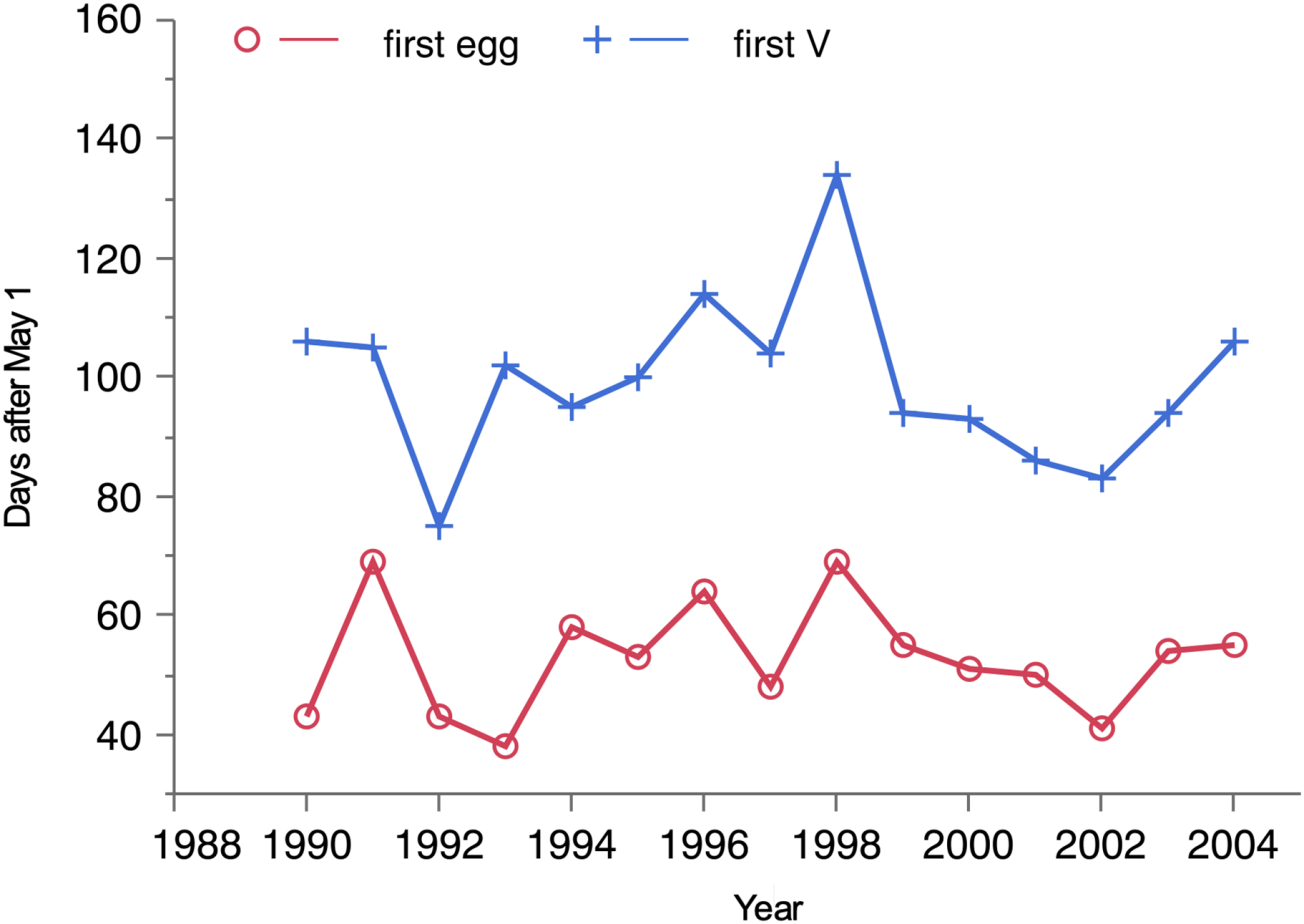
Days (after May 1) of first egg and first last instar larva observed 1990–2004.

**FIGURE 5:**
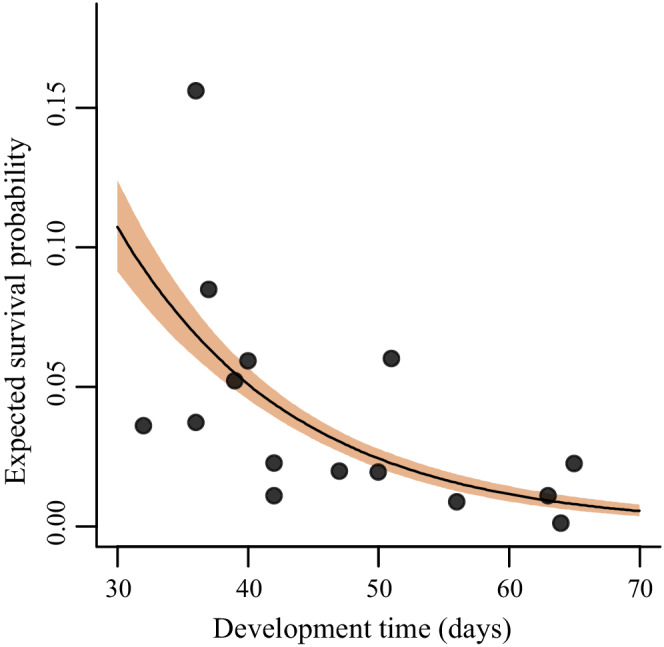
Expected survival probability for the egg–larval period in relation to development time. Calculations made under median value for egg number (374). Shaded area shows 50% intervals.

The duration of the egg–larval development period varied considerably among years (Figure 4). Development time had a strong effect on interval mortality, with higher mortality in summers with slow development (Figure 5, Table 1). The coefficient for development time was estimated to 1.0 (0.6, 1.5), consistent with a constant mortality hazard during larval development. Development time was itself negatively associated with temperature (−4.3 days per degree C, with 95% confidence interval (−7.2, −1.5), *R* ^2^ = .45). In additional models, a direct temperature effect on larval survival was positive when development time was excluded from the model, and indistinguishable from zero when development was included, in line with the hypothesis that temperature affects larval survival mainly via development time (Table 1).

**FIGURE 6:**
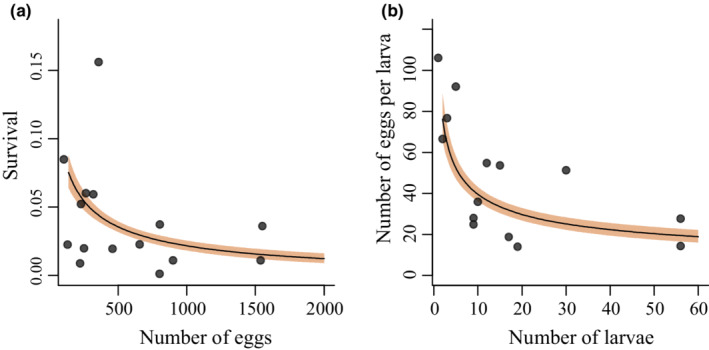
(a) Expected survival probability for the egg–larval period and (b) per capita egg production for the late larva–adult periods in relation to initial densities. In (a), development time has been fixed to its median value (42 days). The shaded areas show 50% intervals.

The coefficient for the density effect of number of eggs on larval survival was negative (Figure 6a, Table 1).

For the period from last instar larvae to eggs in the following year, parameter estimates suggest a density‐dependent pattern in the production of eggs (Figure 6b). There was further a large amount of unexplained variation in the dynamics over this period (Figure 6b, Table 1). Weather effects during this period of the life cycle were uncertain with credible intervals overlapping zero (Table 1).

**FIGURE 7:**
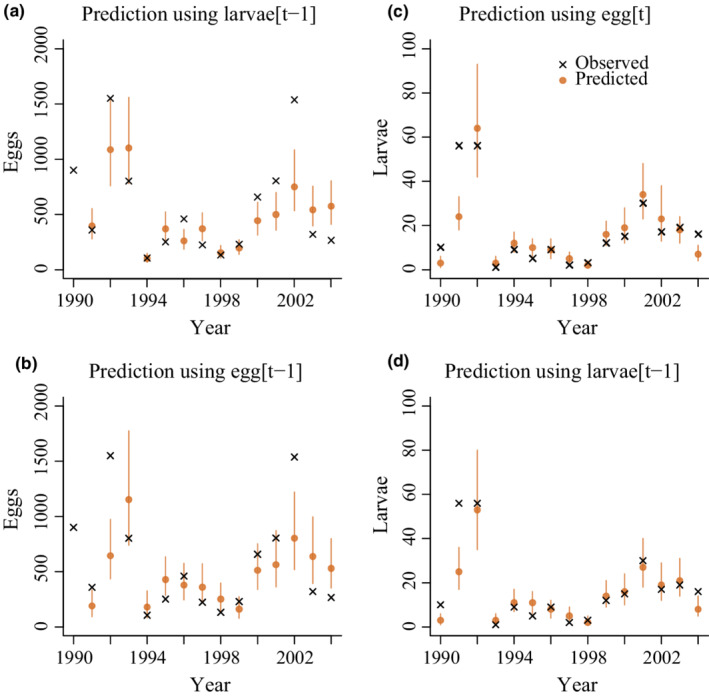
Forward model predictions of egg abundance using (a) the number of larvae in the previous year or (b) the number of eggs in the previous year as the starting point, and of larval abundance using (c) the number of eggs the same year or (d) the number of larvae the previous year. Predictions include observed values of weather variables and larval development times. Lines show 50% prediction intervals.

Comparing forward predictions from the model to observed data (Figure 7) showed that the data were usually within 50% predictive intervals, but with a few observations in the tails of the predictive distribution. Posterior predictive *p*‐values using the mean, standard deviation, minimum, and maximum of the number of eggs and larva as measures of summary statistics did not give evidence of lack of fit (all *p*‐values were within the range from .05 to .95).

**FIGURE 8:**
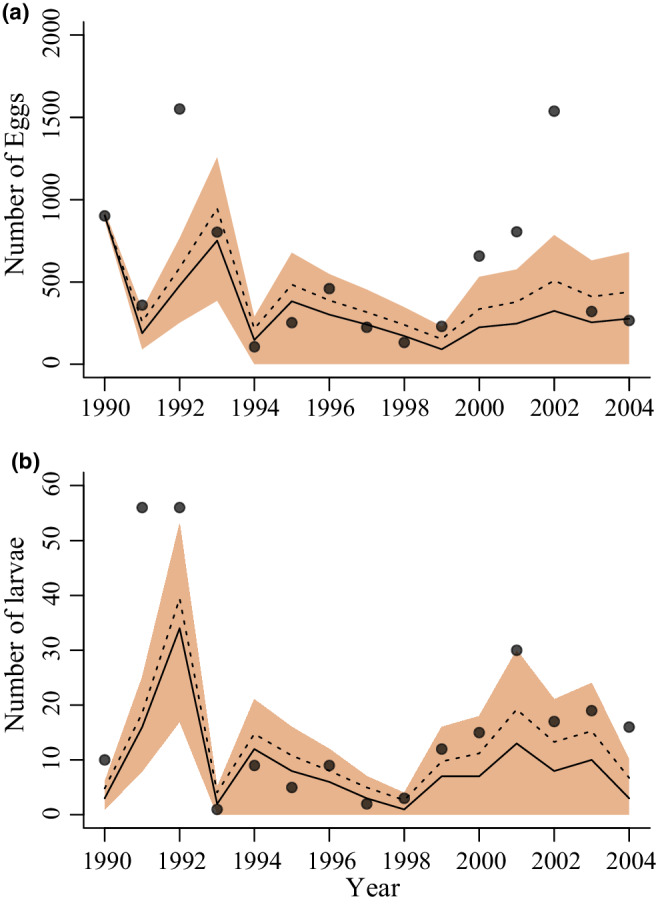
Simulations of (a) egg densities and (b) larval densities from the fitted model. Egg density in 1990 was used to start the simulations. Observed egg–larval development times and weather variables, but not observed egg and larval densities after the starting egg density, were used to propagate the model simulations forward in time. The hatched line gives the mean value and the continuous line the median. The shaded areas show the 50% prediction intervals. Black dots show measured data.

Simulating from the fitted model with only the sequence of weather data and the population start data from 1990 as inputs shows that the model captures essential aspects of population behavior, albeit with considerable variation around medians (or means) (Figure 8), and with populations going extinct in some simulations.

## 4 DISCUSSION

### 4.1 The dynamics of *A. asclepiadis*

A central question with regard to population change is to explore the mechanistic links between population dynamics and climate variability (Boggs & Inouye, 2012; Stenseth et al., 2002). We develop and analyze a model of the dynamics of a noctuid moth population based upon long‐term field data. The life cycle is divided into two phases (1) summer survival of eggs and larvae, and (2) autumn–winter–spring survival followed by spring reproduction. Our study suggests that summer weather effects are important. They seem to be mainly indirect, operating via a window of vulnerability. Slower development of eggs and larvae at lower temperatures leads to an extended exposure to mortality risks. Density‐dependent processes seem to modify these weather‐induced fluctuations. During the rest of the life cycle, there were larger unexplained fluctuations with no clear weather effects.

Many arthropod natural enemies attack the immature stages of *A. asclepiadis*. They are of different sizes and feeding habits, and they all seem to be generalists or at least oligophages (Förare, 1995a, 1995b). A detailed account of egg mortalities (Appendix S1) shows that polyphagous predators form the dominating mortality factor, with a strong contribution by egg parasitoids in certain years (Figure 1a,b).

Several of the egg predators, for example, ants, chrysopid larvae, and anthocorid bugs, also attack and kill young larvae. Larger insect predators, such as pentatomid bugs, attacking the older larvae have often been observed in the plots (Figure 1f). Bird predation is unlikely to be of significance since the later larval stages are night‐active and well concealed during the day.

Parasitoids are likely unimportant for larval mortality as larval parasitoids are very rare in the study area and numerous *A. asclepiadis* larvae brought to the laboratory for other experiments never yielded any parasitoids. Similarly, no diseased larvae were ever found in the field, nor were any sick larvae ever encountered in laboratory rearings (J. Förare, unpublished).

Due to its very low population, density across the landscape *A. asclepiadis* is undoubtedly a minor part in the diets of enemy populations. Thus, it is unlikely that enemy densities are numerically linked to *A. asclepiadis* dynamics. We hence envisage a direct and diffuse pressure by several polyphagous arthropod enemy species, the effect of which is dependent on the length of exposure.

There do not seem to be important *direct* effects of temperature during the egg–larval period. In rearing experiments encompassing a range of naturally encountered temperatures, there were no clear differences in egg or larval mortalities (Förare, 1995a). There are effects of temperature on pupal weight, but they are small for natural conditions (Förare, 1995b). The dominating weather effect on *A. asclepiadis* populations during the summer, therefore, seems to be the indirect effect on the window of vulnerability to enemies. However, as we lack direct data on larval predation, we only have indirect evidence for this conclusion.

The apparent density dependence observed during the egg–larval period is surprising in light of the very low incidence of defoliation observed in the field (Förare, 1995a, 1995b). Although occasional local defoliations have been reported, for example, in Finland (Leimu & Lehtilä, 2006), we have not seen any extensive defoliations during the last 40+ years in our study areas in Sweden. If the statistical density dependence found in our analyses indeed reflects the effects of direct competition, a closer examination of oviposition behavior and conditions at the end of the larval period can solve this apparent paradox. Female moths show an oviposition preference for small and isolated plants in shaded positions (Förare & Engqvist, 1996). Many larvae thus wind up on small individual plants isolated on the scale of a meter or so and may thus experience competition for food on a very local scale (Figure 2g). This effect is strengthened late in summer when leaves start to yellow and fall off. Numerous field observations lend credibility to this idea of small‐scale intraspecific competition for food. Shortage of food for the larvae seems to occur in many populations of Lepidoptera (Dempster, 1983). It need not be due to an absolute shortage of food, but simply a result of an inability of the insect to find it in time (Andrewartha & Birch, 1954). Since measurement error could lead to exaggerated estimates of density dependence, we explore this possibility in Appendix S3, concluding that errors are likely to be fairly small in our study. However, we cannot entirely rule out that they are affecting our density dependence estimates.

Interspecific competition in this *A. asclepiadis* population is highly unlikely since the host plant is poisonous (see above) and no other insects feed on its leaves (except some polyphagous species on rare occasions). Nor do any vertebrates feed on its green leaves.

During the long period (9–10 months) from mature larva in late summer until egg laying in early summer the following year, there remains considerable variation to explain. Almost all of this time is spent as a pupa (inside a cocoon) hidden in the ground. However, the period also involves the final days as a larva and the movement to the pupal site. It also involves the spring period with adult emergence, flight, and oviposition.

We found no clear effects of weather conditions (minimum temperatures or snow conditions) on *A. asclepiadis* during the winter period. This is in contrast to studies of some other Lepidoptera species where winter conditions are important (e.g., Büntgen et al., 2020; Hunter et al., 2014; Roland & Matter, 2016). Nor could we find any clear effect of temperature during spring–early summer when moths emerge, fly, and oviposit.

There is a weak density dependence during the winter, which is of uncertain significance. It could be due to either immigration and/or measurement error (see Appendix S3). In this context, it is interesting that our model points to a considerable risk of local extinction, which however never happened in our plots. This also suggests that extinction‐prone local populations can be “rescued” in spring by migrating moths. However, this is not incorporated in our models due to the lack of explicit data about moth movements.

Predictions of egg number display more unexplained variation than predictions of the number of last instar larvae. Whether this reflects a real difference in predictability or our ignorance of some important process is uncertain. However, it illustrates that the number and specific choice of life history stages can be of importance when analyzing the factors affecting population change.

In general, the lack of precise population data during the wintering period may contribute to the large unexplained variation during this phase of the life cycle.

### 4.2 Idiosyncrasies in the responses of insects to weather factors

What general conclusions concerning insect population dynamics in a new climate can be drawn from our findings about *A. asclepiadis*? The population responses of insects to weather/climate seem to harbor many idiosyncrasies. As mentioned, weather can influence the dynamics of populations via multiple pathways and can affect any stage in the life cycle (e.g., Azerefegne et al., 2001). They can act indirectly via the trophic web and interact with density‐dependent processes. Accordingly, generalizations with regard to weather effects on insect populations are hard to find.

*Abrostola asclepiadis* and the three other insect species feeding on *V. hirundinaria* in the study area add to this list of special cases. The flower gall midge species (*Contarinia vincetoxici* Kieffer) (Cecidomyiidae) fluctuates in multi‐annual cycles with little connection to any direct weather conditions (Solbreck & Widenfalk, 2012). Populations of the seed predatory fly *Euphranta connexa* (Fabr.) (Tephritidae) are strongly coupled to seed density fluctuations, which in turn are determined by both un‐lagged and lagged weather effects (Solbreck & Knape, 2017). Finally, populations of the seed predatory bug *Lygaeus equestris* (L.) (Lygaeidae) show both un‐lagged and moderately lagged responses to direct weather conditions as well as to lagged indirect effects via seed production, but there are no known effects of enemies (Solbreck, 1995). There are thus few commonalities in weather/climate effects in this insect community.

That there are strong idiosyncrasies in responses to weather and climate is further supported when we compare our results to the few previous studies of weather effects on temperate region noctuids. Cool weather resulted in better host plant growth, causing a long‐term plant community–insect density interaction in larval populations of the outbreaking moth *Cerapteryx graminis* (Danell & Ericson, 1990), and an analysis of light trap catches of 12 abundant noctuid moth species in England found that populations were negatively affected by cold and rainy winters (Mutshinda et al., 2011).

The problem of finding simple generalizations pertaining to weather and climate effects on population dynamics is not limited to insects. It seems to be common among many animal groups, as illustrated by a recent review of responses among terrestrial mammals (Paniw et al., 2021). Are we left with a plethora of special cases?

## 5 CONCLUSIONS

We show how weather may affect an insect population indirectly through a temperature‐dependent window of vulnerability toward natural enemies. This process in combination with density‐dependent factors steers population density at the end of the summer egg–larval period. During the remaining part of the yearly life cycle (autumn, winter, and spring), population density is less easily predicted, only to be funneled back toward more easily predicted densities the following summer period. Our study adds yet another mechanism by which weather conditions can affect insect populations.

## AUTHOR CONTRIBUTIONS

**Christer Solbreck:** Conceptualization (equal); data curation (equal); formal analysis (equal); funding acquisition (supporting); investigation (equal); methodology (equal); project administration (lead); resources (supporting); visualization (equal); writing – original draft (equal); writing – review and editing (equal). **Jonas Knape:** Conceptualization (equal); data curation (equal); formal analysis (equal); funding acquisition (supporting); investigation (equal); methodology (lead); software (lead); visualization (equal); writing – original draft (equal); writing – review and editing (equal). **Jonas Förare:** Conceptualization (equal); data curation (equal); investigation (equal); methodology (equal); writing – review and editing (supporting).

## FUNDING INFORMATION

We are grateful for financial support from FORMAS (grant 2017‐01064) to JK.

## Supporting information


Appendix S1

Appendix S2

Appendix S3

Data S1

Data S2

## Data Availability

The data that supports the findings of this study are available in the supplementary material of this article.

## References

[ece39261-bib-0001] Abarca, M. , & Spahn, R. (2021). Direct and indirect effects of altered temperature regimes and phenological mismatches on insect populations. Current Opinion in Insect Science, 47, 67–74. 33989831 10.1016/j.cois.2021.04.008

[ece39261-bib-0002] Ådahl, E. , Lundberg, P. , & Jonzén, N. (2006). From climate change to population change. The need to consider annual life cycles. Global Change Biology, 12, 1627–1633.

[ece39261-bib-0003] Andrewartha, H. G. , & Birch, L. C. (1954). The distribution and abundance of animals. The University of Chicago Press.

[ece39261-bib-0004] Azerefegne, F. , Solbreck, C. , & Ives, A. (2001). Environmental forcing and high amplitude fluctuations in the population dynamics of the tropical butterfly *Acraea acerata* (Lepidoptera: Nymphalidae). The Journal of Animal Ecology, 70, 1032–1045.

[ece39261-bib-0005] Bale, J. S. , Masters, G. J. , Hodkinson, I. D. , Awmack, C. , Bezemer, T. M. , Brown, V. K. , Butterfield, J. , Buse, A. , Coulson, J. C. , Farrar, J. , Good, J. E. G. , Harrington, R. , Hartley, S. , Jones, T. H. , Lindroth, R. L. , Press, M. C. , Symrnioudis, I. , Watt, A. D. , & Whittaker, J. B. (2002). Herbivory in global climate change research. Direct effects of rising temperature on insect herbivores. Global Change Biology, 8, 1–16.

[ece39261-bib-0006] Barton, B. T. , & Ives, A. R. (2014). Species interactions and a chain of indirect effects driven by reduced precipitation. Ecology, 95, 486–494. 24669741 10.1890/13-0044.1

[ece39261-bib-0007] Barton, B. T. , & Schmitz, O. J. (2009). Experimental warming transforms multiple predator effects in grassland food web. Ecology Letters, 12, 1317–1325. 19780788 10.1111/j.1461-0248.2009.01386.x

[ece39261-bib-0008] Benrey, B. , & Denno, R. F. (1997). The slow‐growth‐high‐mortality hypothesis: A test using the cabbage butterfly. Ecology, 78, 987–999.

[ece39261-bib-0009] Boggs, C. L. , & Inouye, D. W. (2012). A single climate driver has direct and indirect effects on insect population dynamics. Ecology Letters, 15, 502–508. 22414183 10.1111/j.1461-0248.2012.01766.x

[ece39261-bib-0010] Büntgen, U. , Liebold, A. , Nievergelt, D. , Wermelinger, B. , Roques, A. , Reinig, F. , Krusic, P. J. , Piermattei, A. , Egli, S. , Cherubini, P. , & Esper, J. (2020). Return of the moth: Rethinking the effect of climate on insect outbreaks. Oecologia, 192, 543–552. 31919693 10.1007/s00442-019-04585-9 PMC7002459

[ece39261-bib-0011] Danell, K. , & Ericson, L. (1990). Dynamic relations between the antler moth and meadow vegetation in northern Sweden. Ecology, 71, 1068–1077.

[ece39261-bib-0012] DeLucia, E. H. , Nabity, P. D. , Zavala, J. A. , & Berenbaum, M. R. (2012). Climate change: Resetting plant‐insect interactions. Plant Physiology, 160, 1677–1685. 22972704 10.1104/pp.112.204750 PMC3510101

[ece39261-bib-0013] Dempster, J. P. (1983). The natural control of populations of butterflies and moths. Biological Reviews, 58, 461–481.

[ece39261-bib-0014] Förare, J. (1995a). Population dynamics of a monophagous insect living on a patchily distributed herb . PhD dissertation, Swedish University of Agricultural Sciences, Uppsala, Sweden.

[ece39261-bib-0015] Förare, J. (1995b). The biology of the noctuid moth *Abrostola asclepiadis* Schiff. (Lepidoptera, Noctuidae) in Sweden. Entomologisk Tidskrift, 116, 179–186.

[ece39261-bib-0016] Förare, J. , & Engqvist, L. (1996). Suboptimal patch and plant choice by an ovipositing monophagous moth – insurance against bad weather? Oikos, 77, 301–330.

[ece39261-bib-0017] Förare, J. , & Solbreck, C. (1997). Population structure of a monophagous moth in a patchy landscape. Ecological Entomology, 22, 256–263.

[ece39261-bib-0018] Hambäck, P. A. (2021). Intra‐ and interspecific density dependence mediates weather effects on the population dynamics of a plant‐insect herbivore system. Oikos, 130, 893–903. 10.1111/oik.08164

[ece39261-bib-0019] Hunter, M. D. , Kozlov, M. V. , Itämies, J. , Pulliainen, E. , Bäck, J. , Kyrö, E.‐M. , & Niemelä, P. (2014). Current temporal trends are counter to predicted effects of climate change in an assemblage of subarctic forest moths. Global Change Biology, 20, 1723–1737. 24421221 10.1111/gcb.12529

[ece39261-bib-0020] Ives, A. R. (1995). Predicting the response of populations to environmental change. Ecology, 76, 926–941.

[ece39261-bib-0021] Kalske, A. , Mutikainen, P. , Muola, A. , Scheepens, J. F. , Laukkanen, L. , Salminen, J.‐P. , & Leimu, R. (2014). Simultaneous inbreeding modifies inbreeding depression in a plant‐herbivore system. Ecology Letters, 17, 229–238. 24304923 10.1111/ele.12223

[ece39261-bib-0022] Kingsolver, J. G. , Woods, A. , Buckley, L. B. , Potter, K. A. , MacLean, H. J. , & Higgins, J. K. (2011). Complex life cycles and the responses of insects to climate change. Integrative and Comparative Biology, 51, 719–732. 21724617 10.1093/icb/icr015

[ece39261-bib-0023] Klapwijk, M. J. , Ayres, M. P. , Battisti, A. , & Larsson, S. (2012). Assessing the impact of climate change on outbreak potential. In P. Barbosa , D. K. Letourneau , & A. A. Agrawal (Eds.), Insect outbreaks revisited (pp. 429–450). Wiley‐Blackwell.

[ece39261-bib-0024] Knape, J. , & de Valpine, P. (2011). Effects of weather and climate on the dynamics of animal population time series. Proceedings of the Royal Society B: Biological Sciences, 278, 985–999. 10.1098/rspb.2010.1333 PMC3049023 20880886

[ece39261-bib-0025] Lawson, C. R. , Vindenes, Y. , Bailey, L. , & van de Pol, M. (2015). Environmental variation and population responses to global change. Ecology Letters, 18, 724–736. 25900148 10.1111/ele.12437

[ece39261-bib-0026] Leimu, R. , & Lehtilä, K. (2006). Effects of two types of herbivores on the population dynamics of a perennial herb. Basic and Applied Ecology, 7, 224–235.

[ece39261-bib-0027] Mutshinda, C. M. , O'Hara, R. B. , & Woiwod, I. P. (2011). A multispecies perspective on ecological impacts of climatic forcing. The Journal of Animal Ecology, 80, 101–107. 20809921 10.1111/j.1365-2656.2010.01743.x

[ece39261-bib-0028] Paniw, M. , James, T. D. , Ruth Archer, C. , Römer, G. , Levin, S. , Compagnoni, A. , Che‐Castaldo, J. , Bennett, J. M. , Mooney, A. , Childs, D. Z. , Ozgul, A. , Jones, O. R. , Burns, J. H. , Beckerman, A. P. , Patwary, A. , Sanchez‐Gassen, N. , Knight, T. M. , & Salguero‐Gómez, R. (2021). The myriad of complex demographic responses of terrestrial mammals to climate change and gaps of knowledge: A global analysis. The Journal of Animal Ecology, 90, 1398–1407. 33825186 10.1111/1365-2656.13467

[ece39261-bib-0029] Pepi, A. , Grof‐Tisza, P. , Holyok, M. , & Karban, R. (2018). As temperature increases, predator attack rate is more important to survival than a smaller window of prey vulnerability. Ecology, 99, 1584–1590. 29672837 10.1002/ecy.2356

[ece39261-bib-0030] Pinder, J. E. , Wiener, J. G. , & Smith, M. H. (1978). The Weibull distribution: A new method of summarizing survivorship data. Ecology, 59, 175–179.

[ece39261-bib-0031] Plummer, M. (2017). JAGS version 4.3.0 user manual . https://kumisystems.dl.sourceforge.net/project/mcmc/jags/Manuals/4.x/jagsusermanual.pdf

[ece39261-bib-0032] Radchuk, V. , Turlure, C. , & Schtickzelle, N. (2013). Each life stage matters: The importance of assessing the response to climate change over the complete life cycle in butterflies. The Journal of Animal Ecology, 82, 275–285. 22924795 10.1111/j.1365-2656.2012.02029.x

[ece39261-bib-0033] Roland, J. , & Matter, S. F. (2016). Pivotal effect of early‐winter temperatures and snowfall on population growth of alpine *Parnassius smintheus* butterflies. Ecological Monographs, 86, 412–428.

[ece39261-bib-0034] Royama, T. (1992). Analytical population dynamics. Chapman and Hall.

[ece39261-bib-0035] Solbreck, C. (1995). Long‐term population dynamics of a seed‐feeding insect in a landscape perspective. In N. Cappuccino & P. W. Price (Eds.), Population dynamics: New approaches and synthesis (pp. 279–301). Academic Press.

[ece39261-bib-0036] Solbreck, C. (2012). Climate change and longterm patch dynamics of a perennial herb. Basic and Applied Ecology, 13, 414–422.

[ece39261-bib-0037] Solbreck, C. , & Knape, J. (2017). Seed production and predation in a changing climate: New roles for resource and seed predator feedback? Ecology, 98, 2301–2311. 28703294 10.1002/ecy.1941

[ece39261-bib-0038] Solbreck, C. , & Widenfalk, O. (2012). Very long diapause and extreme resistance to disturbance in a galling insect. Ecological Entomology, 37, 51–55.

[ece39261-bib-0039] Stenseth, N. C. , Mysterud, A. , Ottersen, G. , Hurrell, J. W. , Chan, K. , & Lima, M. (2002). Ecological effects of climate fluctuations. Science, 297, 1292–1296. 12193777 10.1126/science.1071281

[ece39261-bib-0041] Tullberg, B. , Gamberale‐Stille, G. , & Solbreck, C. (2000). Effects of food plant and group size on predator defence: Differences between two co‐occurring aposematic Lygaeinae bugs. Ecological Entomology, 25, 220–225.

[ece39261-bib-0042] Uszko, W. , Diehl, S. , Englund, G. , & Amarasekare, P. (2017). Effects of warming on predator‐prey interactions – A resource‐based approach and a theoretical synthesis. Ecology Letters, 20, 513–523. 28266168 10.1111/ele.12755

[ece39261-bib-0043] Varley, G. C. , Gradwell, G. R. , & Hassell, M. P. (1973). Insect population ecology an analytical approach. Blackwell.

[ece39261-bib-0044] Walther, G.‐R. (2010). Community and ecosystem responses to recent climatic change. Philosophical Transactions of the Royal Society B: Biological Sciences, 365, 2019–2024. 10.1098/rstb.2010.0021 PMC2880129 20513710

[ece39261-bib-0045] Widenfalk, O. , Gyllenstrand, N. , Sylvén, E. , & Solbreck, C. (2002). Identity and phylogenetic status of two sibling gall midge species (Diptera: Cecidomyiidae: *Contarinia*) on the perennial herb *Vincetoxicum hirundinaria* . Systematic Entomology, 27, 519–528.

