# Supplementary material for: Role of weather and other factors in the dynamics of a low‐density insect population
Source: Ecol Evol. 2022 Sep 6;12(9):e9261. doi: 10.1002/ece3.9261 (PMC9448972; doi:10.1002/ece3.9261)
Supplement: Supplementary file 2 — Appendix S2 [file ECE3-12-e9261-s001.docx]

Appendix 2. Proportion parasitized and predated eggs of *A. asclepiadis* 1990-2004.


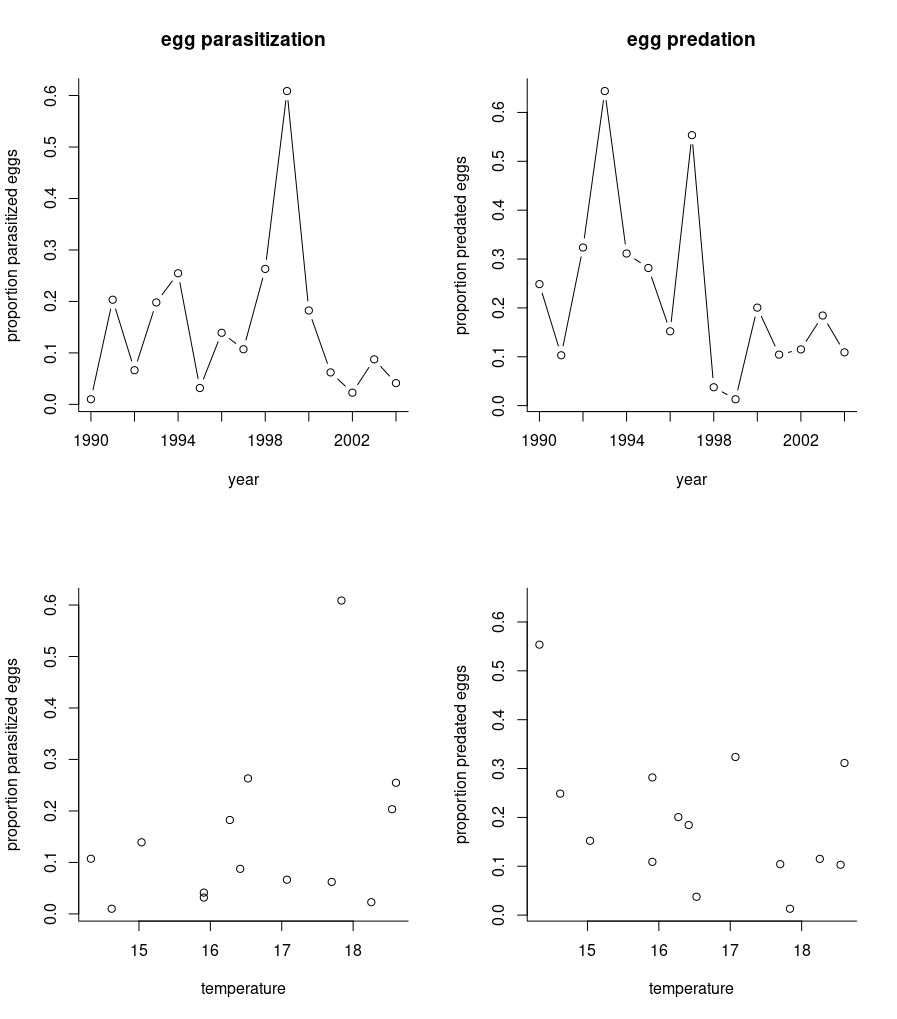
The proportion of parasitized eggs (left column) and proportion of predated eggs (right column) out of all eggs laid as a function of year (top row) and mean temperature over the first 10 days following the first observed egg (bottom row).
