# Supplementary material for: Role of weather and other factors in the dynamics of a low‐density insect population
Source: Ecol Evol. 2022 Sep 6;12(9):e9261. doi: 10.1002/ece3.9261 (PMC9448972; doi:10.1002/ece3.9261)
Supplement: Supplementary file 3 — Appendix S3 [file ECE3-12-e9261-s005.docx]

**Appendix 3. A discussion of measurement error.**

Two measures of abundance are used in our model, namely the number of eggs laid in early summer and the number of last instar larvae produced at the end of the summer season. These numbers are total censuses (not samples) of the plots studied.

The number of **eggs** laid was censused every week by inspecting all host plant leaves in the plots. Each egg clutch was marked and inspected in successive weeks, and the fate of eggs determined as hatched (egg shell with typical larval exit hole), parasitized (darkened egg) or predated. Two types of predator attack eggs namely sucking predators and chewing predators. The first group leaves a shriveled egg shell, whereas the latter either leaves shell remains or eggs disappear (this is of importance when evaluating measurement error, see below).

Because eggs were censused every week, and because eggs or egg shells remain on leaves for more than two weeks, any egg clutch missed on a visit is highly likely to be found in the next week. The error from not finding clutches present on the census days is thus very small. However, one problem remains; egg clutches laid in the interval between two visits may lose eggs without a trace due to predation in the interval.

The number of these unaccounted eggs can be estimated. By taking newly laid egg clutches and looking at the number of eggs disappearing without a trace during the first week this rate of disappearance can be calculated. We analyzed the fate of 434 new egg clutches (from the years 2001-2004). They contained a total of 1557 eggs among which 103 (6,6%) disappeared without a trace. From the above we conclude that egg data are slightly underestimated (never overestimated), but that this rate is usually less that 10%.

The number of last (5th) instar **larvae** was also censused weekly. To aid the search the presence of 4^th^ instar larvae had been mapped in previous weeks, and feeding marks typical of large larvae had also been located in advance. The last instar larvae were censused with the aid of a torch at night, when they are easily found feeding on the top of the plants. All the larvae found were marked so as not to be recounted later.

As last instar larvae usually remain for more than a week any missed larvae are likely found on later visits. The degree of underestimate connected to larval censuses is hard to estimate but probably quite small. What is probably more important for our analysis is that the number of larvae is very small in absolute terms (1-56). This means that stochastic effects may be large. Small errors in absolute terms may have a large effect on estimates of density dependence.

Another important aspect in this connection is the role of movements. Immature stages are confined to the studied areas. However, adult moths are good fliers (Förare & Solbreck 1997) frequently moving between different host plant patches during the oviposition period. The number of eggs deposited is thus affected by the general density of moths on the landscape level. If the number of larvae is low due to an underestimate and/or as a result of local random effects, immigration will have a stronger effect and hence appear density dependent.

We have an index of larval densities based upon feeding damage (Förare & Solbreck 1997) (for data see appendix 1 this paper) for most host plant patches in the study area for the years in question (Solbreck unpubl.). The proportion of this index density (= general density of moths on the landscape scale) vs density of last instar larvae in our plots shows a strong increase as plot larval density decreases (Fig. 1). This suggests that immigration is more important (density dependent) when local density is low. An alternative explanation is that density dependence is spurious due to measurement errors.


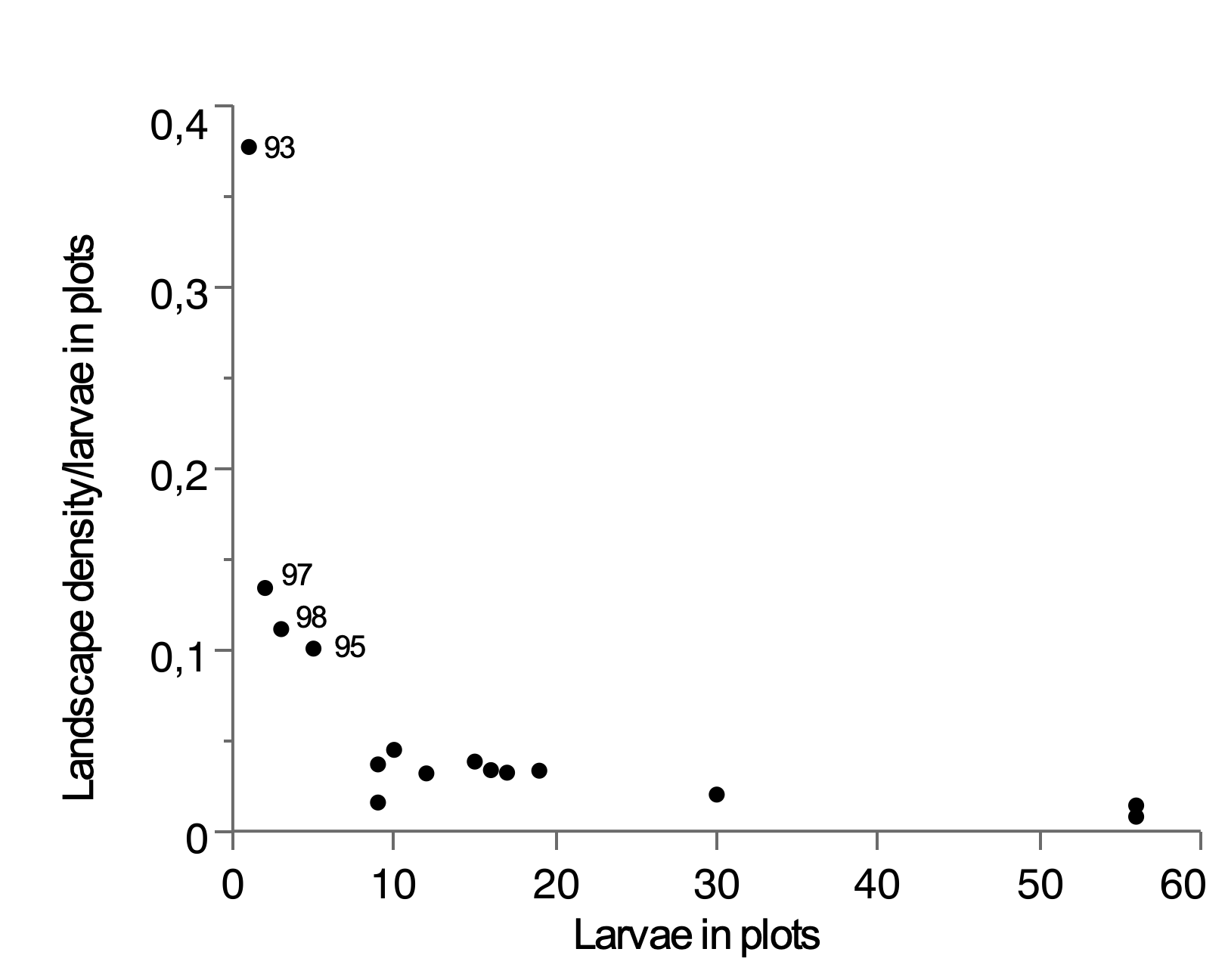


Fig. 1. Index for larval density on landscape scale in relation to larval density in plots vs larval density in plots. Numbers next to points indicate years.
